# Amniotic MSCs reduce pulmonary fibrosis by hampering lung B‐cell recruitment, retention, and maturation

**DOI:** 10.1002/sctm.20-0068

**Published:** 2020-05-26

**Authors:** Anna Cargnoni, Pietro Romele, Patrizia Bonassi Signoroni, Serafina Farigu, Marta Magatti, Elsa Vertua, Ivan Toschi, Valentina Cesari, Antonietta R. Silini, Francesca R. Stefani, Ornella Parolini

**Affiliations:** ^1^ Centro di Ricerca E Menni, Fondazione Poliambulanza‐Istituto Ospedaliero Brescia Italy; ^2^ Dip. Scienze Agrarie e Ambientali Università degli Studi di Milano Milan Italy; ^3^ Department of Life Science and Public Health Università Cattolica del Sacro Cuore Roma Italy

**Keywords:** amniotic mesenchymal stromal cells, B lymphocytes, bleomycin, lung fibrosis

## Abstract

Growing evidence suggests a mechanistic link between inflammation and the development and progression of fibrotic processes. Mesenchymal stromal cells derived from the human amniotic membrane (hAMSCs), which display marked immunomodulatory properties, have been shown to reduce bleomycin‐induced lung fibrosis in mice, possibly by creating a microenvironment able to limit the evolution of chronic inflammation to fibrosis. However, the ability of hAMSCs to modulate immune cells involved in bleomycin‐induced pulmonary inflammation has yet to be elucidated. Herein, we conducted a longitudinal study of the effects of hAMSCs on alveolar and lung immune cell populations upon bleomycin challenge. Immune cells collected through bronchoalveolar lavage were examined by flow cytometry, and lung tissues were used to study gene expression of markers associated with different immune cell types. We observed that hAMSCs increased lung expression of T regulatory cell marker Foxp3, increased macrophage polarization toward an anti‐inflammatory phenotype (M2), and reduced the antigen‐presentation potential of macrophages and dendritic cells. For the first time, we demonstrate that hAMSCs markedly reduce pulmonary B‐cell recruitment, retention, and maturation, and counteract the formation and expansion of intrapulmonary lymphoid aggregates. Thus, hAMSCs may hamper the self‐maintaining inflammatory condition promoted by B cells that continuously act as antigen presenting cells for proximal T lymphocytes in injured lungs. By modulating B‐cell response, hAMSCs may contribute to blunting of the chronicization of lung inflammatory processes with a consequent reduction of the progression of the fibrotic lesion.

## Graphical abstract

This study demonstrates that amniotic mesenchymal stromal cells reduce pulmonary fibrosis in bleomycin‐challenged mice by creating an anti‐inflammatory microenvironment mediated by their ability to control B‐cell recruitment, retention, maturation, and to reduce the formation/expansion of lymphoid aggregates in diseased lungs.

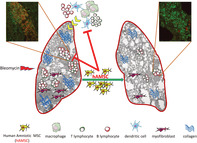

**Significance statement**

The immunomodulatory features of amniotic cells can create a microenvironment able to limit the evolution of chronic inflammation to fibrosis. However, the immune modulation induced by amniotic mesenchymal stromal cells (hAMSCs) in models of fibrosis has yet to be elucidated. For the first time, this study shows that in bleomycin‐challenged mice, hAMSCs control pulmonary B‐cell recruitment, retention, maturation, and reduce the formation and expansion of lung lymphoid aggregates. By modulating B‐cell response, hAMSCs hamper the self‐maintaining inflammatory condition promoted by B cells in injured lungs and may contribute to limiting the chronicization of lung inflammation that evolves into the fibrotic lesion.

## 1 INTRODUCTION

The pathogenesis of idiopathic pulmonary fibrosis (IPF) is still poorly understood, thus heavily contributing to the lack of an effective cure.

There is growing evidence suggesting a mechanistic link between inflammation, which endures to the end stage of IPF, and the development and progression of fibrosis.^1^, ^2^, ^3^, ^4^

Interestingly, we and others have reported that cells derived from the amniotic membrane of human term placenta, such as amniotic epithelial cells and amniotic mesenchymal stromal cells (hAMSCs)^5^, ^6^, ^7^, ^8^, ^9^, ^10^ and related secretome,^11^, ^12^, ^13^ can prevent and reduce the progression of pulmonary fibrosis when injected in bleomycin‐instilled rodents, a model which closely resembles human IPF.^14^

Amniotic cells and their secreted factors possess in vitro and in vivo immune‐modulatory properties. They inhibit T‐cell proliferation,^15^, ^16^, ^17^, ^18^ reduce the production of proinflammatory cytokines,^6^, ^7^, ^18^, ^19^ block the differentiation/maturation of monocytes into dendritic cells,^20^, ^21^, ^22^ promote macrophage polarization toward an M2 anti‐inflammatory phenotype,^8^, ^23^, ^24^ induce regulatory T cells,^18^, ^25^, ^26^ and reduce natural killer cytotoxicity.^27^

However, to the best of our knowledge, no studies investigated the crosstalk between hAMSCs and immune cell populations that are involved in bleomycin‐induced lung injury.

Herein, we sought to explore whether hAMSC treatment affects the levels and phenotype of immune cell populations in alveolar spaces and in lung tissue during the course of bleomycin‐induced lung injury, and its potential correlation to therapeutic outcome.

Furthermore, in an attempt to address the important question about the impact of in vitro culture on hAMSC therapeutic effects, in this study we used both nonexpanded passage 0 hAMSCs (hAMSC/P0) and hAMSCs expanded in vitro to passage 2 (hAMSC/P2).

## 2 MATERIALS AND METHODS

### 2.1 Ethics statement

Human term placentae were collected from healthy subjects, according to the guidelines of the Ethical Committee of the Province of Brescia (Comitato Etico Provinciale, approval CEP 19.01.2016).

Animal experiments were carried out in accordance with the guidelines established by the Italian law DL 26/2014. The experimental protocol was approved by Italian Ministry of Health (Authorization n. 779/2016‐PR) and by the Committee on the Ethics of Animal Experiments of the University of Milano (n. 105/14, 9/12/2014).

### 2.2 hAMSCs isolation, culture, and characterization

Twelve amniotic membranes were collected and processed as previously described.^28^ Briefly, the amniotic membrane was manually separated from the chorion, rinsed in saline solution containing 100 U/mL penicillin and 100 μg/mL streptomycin (both from Sigma‐Aldrich, St. Louis, Missouri), fragmented (~3 × 3 cm^2^) and then digested at 37°C with 2.5 U/mL dispase (Corning, New York). After 9 minutes, the amnion was washed in RPMI 1640 medium (Sigma‐Aldrich) supplemented with 10% fetal bovine serum (FBS; Sigma), 2 mM L‐glutamine (Sigma‐Aldrich), and P/S (herein referred to as complete RPMI medium), and finally digested for 2.5 hours with 0.94 mg/mL collagenase and 0.01 mg/mL DNase (both from Roche, Basel, Switzerland). Isolated cells were filtered (100 μm strainer, BD Falcon, Bedford, Massachusetts), collected by centrifugation, resuspended in complete RPMI medium, and refiltered (70 μm strainer BD Falcon). All cell batches were used as individual preparations that were cryopreserved and/or in vitro cultured.

Freshly isolated cells (hAMSC/P0) were frozen in 90% FBS and 10% dimethyl sulfoxide (DMSO; Sigma‐Aldrich) and stored in liquid nitrogen until further use. Alternatively, isolated cells (hAMSC/P0) were plated at a density of 10 000 cells/cm^2^ in CHANG Medium‐C (Irvine Scientific, Santa Ana, California) supplemented with 2 mM L‐glutamine and P/S. When reaching 80% confluence, cells were subcultured at a density of 10 000 cells/cm^2^. Expanded hAMSCs (passage 2; hAMSC/P2), were detached and frozen as described above.

Both hAMSC/P0 and hAMSC/P2 were phenotypically characterized for the expression of mesenchymal (CD105, CD90, CD73, CD13), hematopoietic (CD45) and epithelial (CD326) markers by flow cytometry (FACS Calibur cytometer), as previously reported.^21^ The following antibodies were used: anti‐CD90 (1:500, clone 5E10); anti‐CD73 (1:50, clone AD2), anti‐CD13 (1:500, clone L138), anti‐CD45 (1:250, clone 2D1), and anti‐CD326 (1:50, clone EBA‐1) all from BD Biosciences (San Jose, California); and anti CD105 (1:50, clone SN6 from Serotec, Oxford, UK).

Cells whose viability after thawing was higher than 85% were used. Nonexpanded and in vitro expanded cell preparations were used in order to study the possible impact of in vitro culture on hAMSC therapeutic effects.

### 2.3 Induction of lung injury and treatment

All experimental procedures were performed on animals anesthetized via intramuscular injection with Tiletamine chloride + zolazepam chloride (Zoletil‐100, Virbac, Milano, Italy, at 0.3 mL/30 mg/kg) and xylazine (Ronpum, Bayer Schering Pharma AG, Leverkusen, Germany, at 2 mg/kg).

Lung injury was induced by intratracheal (i.t.) instillation of bleomycin (40 μL; 2.3 U/kg; Bleoprim, Sanofi‐Aventis, Milano) in 8‐ to 9‐week‐old female C57BL/6 mice (Charles River, Calco, Italy).^5^ Healthy control animals received 40 μL of saline.

Fifteen minutes after treatment with bleomycin or saline, 1 × 10^6^ hAMSC/P0 or hAMSC/P2 were injected i.t., as previously described.^5^ Control animals were injected with only 150 μL sterile PBS.

Each treatment group (Bleo+PBS, Bleo+hAMSC/P0, and Bleo+hAMSC/P2) consisted of n = 26 animals. Notably, each treatment group was composed of five different time point subgroups: day 2 (n = 3); day 4 (n = 4); day 7 (n = 5); day 9 (n = 7), and day 14 (n = 7). The size of each treatment/time point group is indicated in each figure.

### 2.4 Sample collection

Each animal was anesthetized as described above and euthanized at the scheduled time point by exsanguination. Blood was drained from the abdominal aorta and bronchoalveolar lavage (BAL) was collected using a total volume of 2.0 mL of sterile PBS injected i.t. with a 20‐gauge cannula. BAL fluids were centrifuged (1000*g* for 10 minutes, at 4°C) and cells were frozen in 90% FBS+10% DMSO for flow cytometry analysis.

Lungs were explanted and sectioned into the five individual lobes as previously described.^12^ Each lobe was further sectioned into two equivalent hemilobes. One series of hemilobes was formalin‐fixed (10% neutral formalin from Bio‐Optica, Milano, Italy) for 48 hours at room temperature and processed for microscopic analyses. The other series of pooled hemilobes was snap‐frozen in liquid nitrogen and stored at −80°C for real‐time polymerase chain reaction (RT‐PCR) analysis.

### 2.5 Microscopy and image analysis

Lung hemilobes were paraffin‐embedded and consecutive 4‐μm‐thick sections were cut. Sequential, nonoverlapping images were captured from whole hematoxylin and eosin or Masson's trichrome‐stained sections with a digital camera (Olympus Camedia C‐4040 ZOOM) in bright‐field light microscopy (Olympus BX41, Tokyo, Japan) at 40× magnification. Color digital images obtained from each hemilobe were converted by the FiJi software (https://imagej.nih.gov/ij) to binary data, and the percentage of each alveolar hemilobe pixels to whole hemilobe pixels was calculated. The area occupied by alveoli of the entire lung was the sum of all hemilobe alveolar areas and was expressed as a percentage of total area of the entire lung section.^9^, ^29^ All analyses were performed in a blinded manner by a veterinary pathologist.

### 2.6 Flow cytometry analysis

BAL cells were stained with Zombie NIR Live/Dead Cell Kit (eBiosciences, San Diego, California) for live/dead discrimination according the manufacturer's instructions. After 5 minutes incubation with CD16/CD32 (Mouse Fc Block, BD Biosciences), cells were stained for 20 minutes at 4°C with the following anti‐mouse antibodies: CD45 FITC (1:1000, 553080 clone 30‐F11), CD3e PE (1:160, 553063 clone 145‐2C11); CD4 BV421 (1:2000, 740007 clone RM4‐5), CD8a BV510 (1:160, 563068 clone 53‐6.7), CD25 PE‐CF594 (1:100, 562694 clone PC61), B220 PerCP‐Cy5.5 (1:500, 561101 clone RA3‐6B2), CD19 PE‐Cy7 (1:100, 552854 clone 1D3), CD11b BV421 (1:200, 562605 clone M1/70), CD11c PE‐Cy7 (1:100, 558079 clone HL3), I‐A/I‐E (MHC‐II) BV510 (1:330, 742893 clone M5/114.15.2), CD24 APC (1:2000, 562349 clone M1/69), CD64 PE (1:500, 558455 clone X54‐5/7.1), Siglec‐F PE‐CF594 (1:200, 562757 clone E50‐2440), and CD80 BV510 (1:100, 740130 clone 16‐10A1; all from BD Biosciences).

In order to detect intracellular expression of FoxP3, cells were fixed and permeabilized with Cytofix/cytoperm solution (BD Biosciences; 20 minutes, 4°C) and subsequently incubated with anti‐mouse FoxP3 A647 (1:200, 563486 clone R16‐715; BD Biosciences) for 30 minutes at 4°C.

Antigen expression was detected using BD FACSAria III equipped with the BDFACSDiva software (BD Biosciences) and data were analyzed with the FCSExpress 5.0 software (DeNovo Software, Los Angeles, California).

Cell populations were identified by sequential gating strategy following previously published protocols^30^, ^31^, ^32^ with modifications. Briefly, cells were identified as follows: neutrophils (CD11b^+^ CD11c^−^ CD24^+^ Siglec‐F^−^); alveolar macrophages (CD64^+^ CD24^−^ CD11c^+^ CD11b^−^ Siglec‐F^+^); monocyte‐derived alveolar macrophages (CD64^+^ CD24^−^ CD11c^+^ CD11b^+^ Siglec‐F^low/−^); dendritic cells CD11b^−^ (CD64^−^ CD24^+^ I‐A/I‐E^+^ Siglec‐F^−^ CD11b^−^); dendritic cells CD11b^+^ (CD64^−^ CD24^+^ I‐A/I‐E^+^ Siglec‐F^−^ CD11b^+^); B lymphocytes (CD3^−^ B220^+^ CD19^+^); T lymphocytes (CD3^+^ CD4^+^ and CD3^+^ CD8^+^); regulatory T cells (CD3^+^ CD4^+^ CD25^+^ FoxP3^+^). The gating strategy applied for cell identification is reported in Supporting Information Figure S1. All cells contained within the BAL collected from each animal were analyzed. At least 20 000 events for each BAL sample were acquired after surface or intracellular staining.

Results are presented as percentage of viable cells. The CD80 marker is expressed as median fluorescence intensity ratio between positive and negative cells. Specific isotype controls were used.

### 2.7 Quantitative RT‐PCR

Gene expression in lung tissue of podoplanin, α‐SMA, fibronectin, and collagen was determined by RT‐PCR as follows. Total RNA was extracted from snap‐frozen lung hemilobes using EZ1 RNA Universal Tissue Kit (Qiagen, Hilden, Germany) following manufacturer's instructions. cDNA was prepared with ImProm‐IITM Reverse Transcription System (Promega, Madison, Wisconsin). Quantitative RT‐PCR was performed using PowerUp Green Master Mix SYBR Green PCR master kit (Life Technologies) on a ABIPRISM 7000 instrument (Applied Biosystems). Data were normalized to the housekeeping gene (β‐actin) and relative gene expression was quantified using the 2^−ΔCT^ method. Primer sequences are shown in Supporting Information Table S1.

### 2.8 Immunofluorescence and quantification of lymphoid aggregates

CD3^+^ T, B220^+^ B, and CD138^+^ antibody‐secreting cells were identified by immunofluorescence as follows. Paraffin‐embedded sections of lung tissue (4 μm) were de‐waxed and rehydrated in water. Antigen retrieval was performed with 10 mM citrate buffer, pH 8.0 (W‐CAP from Bio‐Optica, Milano, Italy) at 98°C for 10 minutes. After blocking (10% normal goat serum, Thermo Fisher Scientific, San Diego, California), sections were incubated with polyclonal rabbit anti‐CD3 antibody (1:20, Dako, Glostrup, Denmark) and rat anti‐CD45R/B220 antibody (1:100, Clone RA3‐6B2, Thermo Fisher Scientific) or polyclonal rabbit anti‐CD138 antibody (1:50, Thermo Fisher Scientific) at 4°C O/N. After washing, sections were incubated with secondary Cy5 anti‐rat antibody (5 μg/mL, Vector Laboratories, Inc, Burlingame, California) or biotinylated anti‐rabbit antibody (IgG(H+L), Vector Laboratories), followed by DyLight 488 Streptavidin (2 μg/mL, Vector Laboratories). Images were acquired using the Nikon Eclipse Ni‐U microscope equipped with Mono Camera Nikon DS‐Fi3 Version 4.60.

Lymphoid aggregates were counted in two sections of all lung hemilobes by CD3/B220 expression. The size of each aggregate was measured using the FiJi software and the cumulative area occupied by lymphoid aggregates was calculated as the sum of all individual areas present in all lung hemilobes per section. Lymphoid aggregates were stratified according to their size (small: 5000‐10 000 μm^2^; intermediate: 10 000‐20 000 μm^2^; large: >20 000 μm^2^), and proportion of B cells with respect to the whole lymphoid population (low: 0%‐10%; intermediate: 10%‐40%; large: 41%‐70%).

### 2.9 Statistical analysis

All data are presented as mean ± SE of three independent experiments. Differences between groups were analyzed by one‐way analysis of variance, followed by Tukey's multiple comparisons test. A *P*‐value <.05 was considered statistically significant. Statistical analyses were performed using the Prism 6.05 software (Graphpad software Inc., La Jolla, California).

## 3 RESULTS

### 3.1 hAMSCs ameliorate bleomycin‐induced lung injury

To explore the potential benefits exerted by hAMSCs, we evaluated bleomycin‐induced lung injury by measuring residual alveolar area and gene expression of markers indicative of alveolar integrity (eg, podoplanin)^33^ and associated with progression of fibrotic lesions (eg, α‐SMA, fibronectin, and collagen).

**FIGURE 1:**
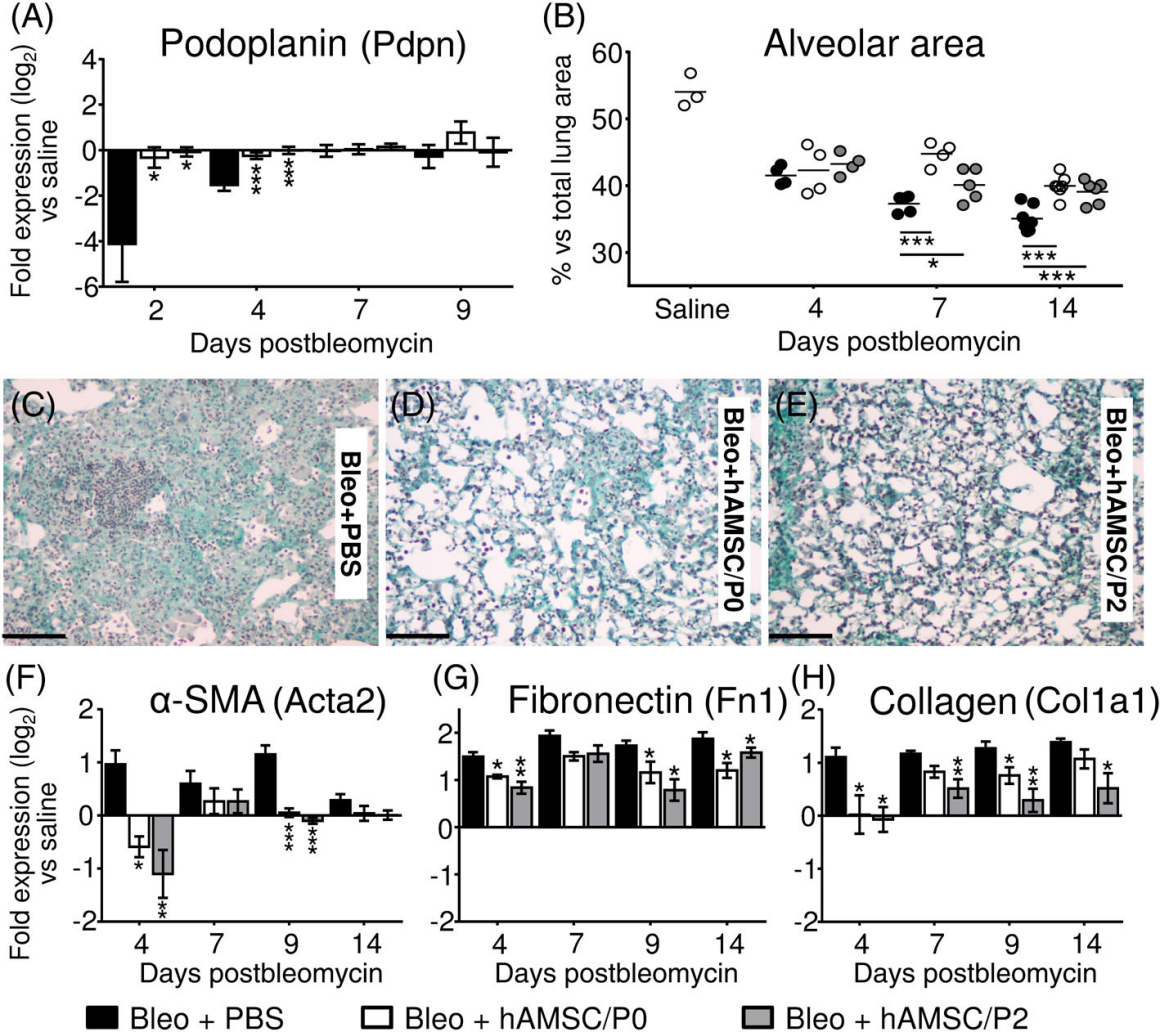
Human amniotic mesenchymal stromal cells (hAMSCs) ameliorate bleomycin‐induced lung injury. Compared to saline, bleomycin instillation (black bars) produced a marked drop of podoplanin expression (a marker of type I alveolar epithelial cells), (A, Bleo+PBS group) and a progressive reduction of lung alveolar area (B, Bleo+PBS group), due to interstitial septa thickening and alveolar obliteration (C, Bleo+PBS group). Moreover, bleomycin challenge increased lung expression of α‐SMA (a marker of myofibroblasts), (F, Bleo+PBS group), and of extracellular matrix proteins such as fibronectin (G, Bleo+PBS group) and collagen (H, Bleo+PBS group). Treatment with hAMSCs (at passage 0 = hAMSC/P0, white bars or at passage 2 = hAMSC/P2, gray bars) preserved lung gene expression of podoplanin (A) and alveolar area (B), and decreased alveolar obliteration (D,E). Moreover, hAMSCs reduced lung gene expression of α‐SMA (F), fibronectin (G), and collagen (H). Data from mRNA levels analysis are expressed as log_2_ of the fold change from the saline‐instilled group. These data are reported as mean ± SE of n = 3 (day 2); n = 4 (day 4) and n = 5‐7 (days 7, 9, and 14) samples. Microphotographs are representative of lung tissues collected from Bleo+PBS group (C), Bleo+hAMSC/P0 (D), and Bleo+hAMSC/P2 (E) at day 14 postbleomycin. Scale bars = 100 μm. *P < .05, **P < .01, ***P < .001 compared to control (Bleo+PBS) group

Alveolar epithelial damage occurred early after bleomycin instillation (at day 2) as shown by marked decrease of lung podoplanin gene expression by 84.2% ± 0.1% (Figure 1A).

At day 14 postinstillation, the alveolar area assessed in animals of Bleo+PBS group declined (from 54.0 ± 2.5 to 35.1 ± 0.7 in saline and bleomycin‐instilled mice, respectively; *P* < .01; Figure 1B) as a result of a progressive interstitial septa thickening and alveolar obliteration (Figure 1C).

Treatment with hAMSCs preserved lung expression of podoplanin at days 2 and 4 (Figure 1A), as well as better maintained residual alveolar area (Figure 1B) with decreased alveolar obliteration (Figure 1D,E).

In addition, hAMSCs induced a significant reduction of lung gene expression of α‐SMA (a myofibroblast marker) at days 4 and 9 (Figure 1F), and a decrease in expression of extracellular matrix proteins such as fibronectin (Figure 1G) and collagen (Figure 1H) occurred at all time points.

### 3.2 hAMSCs alter antigen presenting cells in bleomycin‐induced inflammation

To investigate a possible link between the immune‐modulatory properties of hAMSCs and their ability to limit the progression of lung fibrosis, we studied the capacity of hAMSCs to reduce bleomycin‐induced recruitment of inflammatory cells into alveolar spaces and lung tissue, as well as their polarization and maturation.

Bleomycin challenge induced a recruitment of inflammatory cells into alveolar spaces. hAMSC treatment did not affect the alveolar recruitment of CD45^+^ cells at any time point (see Supporting Information Table S2); however, relative differences among CD45^+^ cell subpopulations were observed.

**FIGURE 2:**
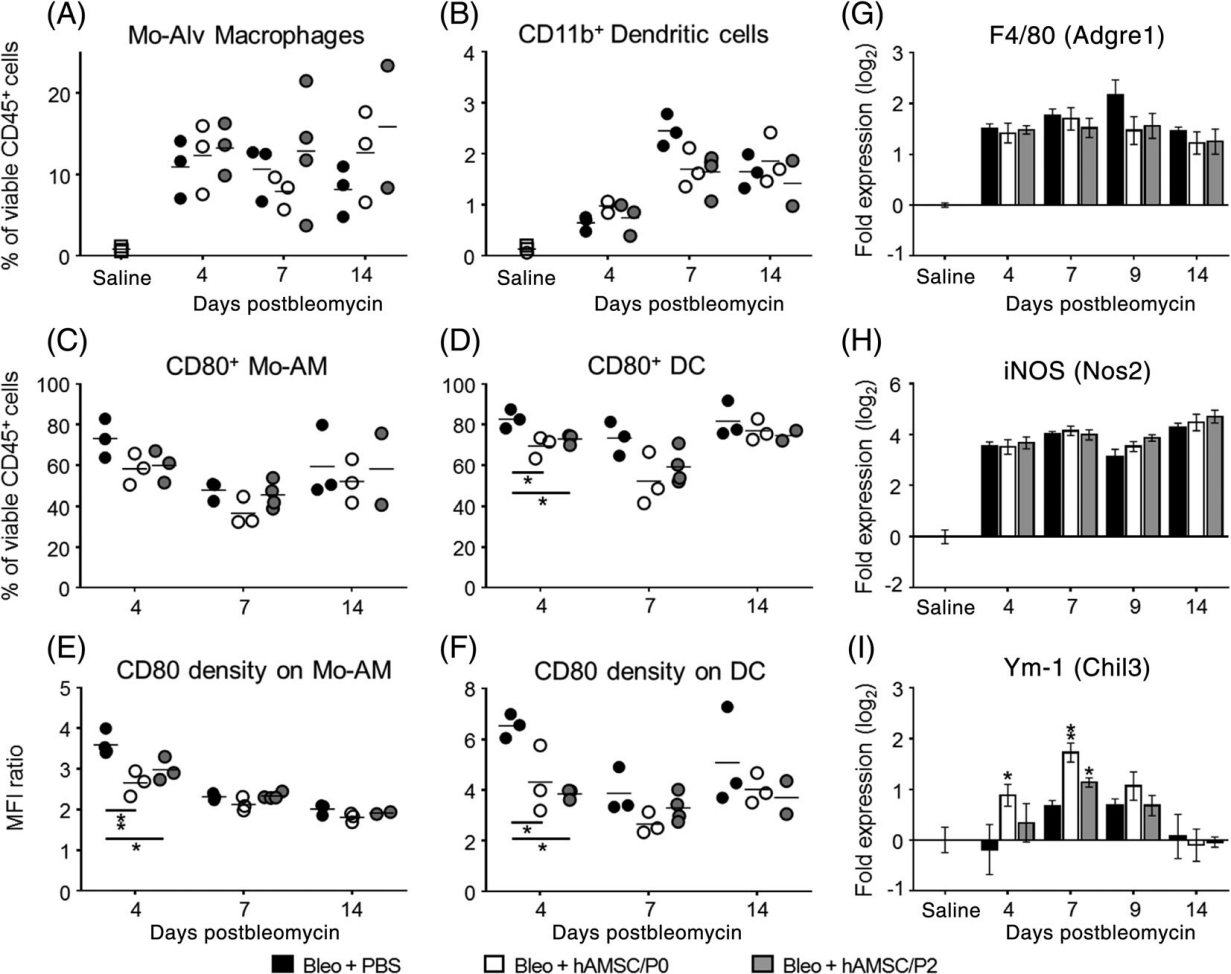
Human amniotic mesenchymal stromal cells (hAMSCs) alter antigen presenting cells in bleomycin‐induced inflammation. Bleomycin challenge increased alveolar levels of both monocyte‐derived alveolar macrophages (Mo‐Av Macrophages, Mo‐AM) (A, Bleo+PBS group) and CD11b^+^ dendritic cells (DC) (B, Bleo+PBS group). hAMSC treatment did not affect levels of Mo‐AM and DC (A,B), however it reduced the levels of Mo‐AM and DC expressing the costimulatory molecule CD80 (C,D) and more strongly reduced the density with which CD80 was expressed on the surface of these cells (E,F). With regard to macrophage subsets, bleomycin challenge increased lung mRNA expression of F4/80 (a pan marker of macrophages; G, Bleo+PBS group) and of iNOS (a marker of M1 macrophages; H, Bleo+PBS group) while did not change expression of Ym‐1 (a marker of M2 macrophages, I). hAMSC treatment did not affect expressions of F4/80 and iNOS, in contrast it increased the expression of Ym‐1 (I), suggesting a polarization toward M2 anti‐inflammatory phenotype. Alveolar levels of Mo‐AM and DC were analyzed by flow cytometry, as percentage of viable CD45 positive cells in bronchoalveolar lavage collected from bleomycin‐challenged mice treated with amniotic cells (Bleo+hAMSC/P0 and Bleo+hAMSC/P2) or not (Bleo+PBS). Density of CD80 expression was evaluated as median fluorescence intensity ratio between positive and negative cells. Data from mRNA levels analysis are expressed as log_2_ of the fold change from the saline‐instilled group. These data are reported as mean ± SE of n = 4 (day 4) and n = 5‐7 (days 7, 9, and 14) samples. *P < .05, **P < .01 compared to control (Bleo+PBS) group

Bleomycin instillation produced marked alterations of immune populations present in alveolar spaces. In bleomycin‐instilled animals, we observed a marked decline of relative amount of resident alveolar macrophages, identified as CD64^+^ CD11c^+^ Siglec‐F^+^ cells, which represent the predominant cell population in normal conditions. Their percentage fell from 78.2% ± 2.4% in the saline control group, to 41.8% ± 5.8% and 8.6% ± 2.9% after 4 and 7 days from bleomycin instillation, respectively. In contrast, the percentage of not resident monocyte‐derived macrophages (identified as CD64^+^ CD11c^+^ Siglec‐F^low/−^ cells and herein referred to as Mo‐Alv macrophages) increased from 0.8% ± 0.3% in saline‐instilled mice to 10.9% ± 3.4% at day 4 postbleomycin and maintained stable up to day 14 (Figure 2A). It is of note that a large percentage of Mo‐Alv macrophages (78.5% ± 13.1%) expressed the costimulatory molecule CD80, in contrast with the low percentage (12.8% ± 5.8%) of CD80‐expressing resident alveolar macrophages. Given the crucial role played by CD80 in triggering T‐cell mediated antigen‐specific inflammatory responses, this finding suggests that bleomycin promotes the recruitment of Mo‐Alv macrophages which can act as antigen presenting cells more efficiently than resident alveolar macrophages.

Similar to Mo‐Alv macrophages, alveolar percentage of dendritic cells, another immune population with crucial antigen presenting activity, increased from 0.12% ± 0.06% in saline‐instilled mice to 2.45% ± 0.31% at day 7 postbleomycin (Figure 2B). Dendritic cells have been identified as CD11b^+^ MHCII^+^ CD11c^+^ CD24^−^ CD64^−^ cells, and herein referred to as CD11b^+^ DCs (Figure 2B).

**FIGURE 3:**
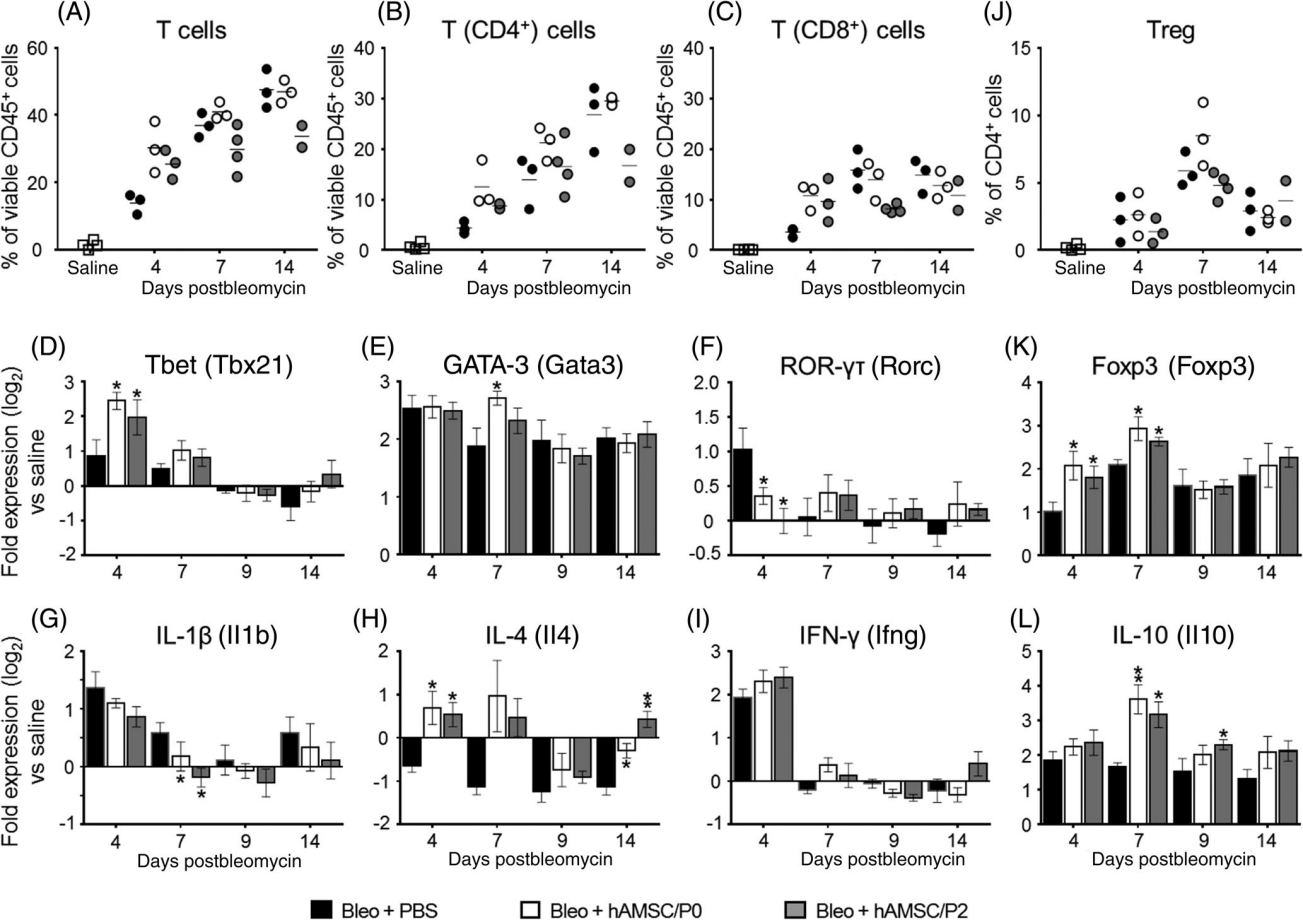
Human amniotic mesenchymal stromal cells (hAMSCs) alter T lymphocyte subsets in bleomycin‐induced inflammation. Bleomycin instillation produced recruitment of T lymphocytes in alveolar spaces (A, Bleo+PBS group), and increased both CD4^+^ and CD8^+^ T‐cell subpopulations (B,C, Bleo+PBS group). hAMSC treatment slightly and transiently increased T lymphocyte recruitment (A‐C). Panels D‐F report lung mRNA levels of markers associated with distinct subsets of T lymphocytes including Tbet (related to Th1 cells), GATA3 (Th2), and ROR‐γτ (Th17). Bleomycin challenge increased the expression of these markers (D‐F, Bleo+PBS group). hAMSC treatment transiently increased expression of Tbet (D, day 4) and of GATA3 (E, day 7), while reducing ROR‐γτ gene expression (F, day 4). Panels G‐I report gene lung expressions of cytokines secreted by Th1 T‐cell subset (IL‐1β and IFN‐γ, G,H) and Th2 subset (IL‐4, I). Bleomycin challenge increased expressions of IL‐1β and IFN‐γ (G,H, Bleo‐PBS group), except IL‐4 which is reduced compared to saline instillation (I, Bleo+PBS group). Treatment with hAMSCs did not change lung expression of IFN‐γ (G), reduced that of IL‐1β (H), and increased that of IL‐4 (I), suggesting a polarization toward Th2 T‐cell subset. Panels J‐L show alveolar levels of T regulatory (Treg) cells and the expression of markers related to an anti‐inflammatory milieu. Compared to saline‐instilled group, animals challenged with bleomycin displayed increased levels of alveolar Treg (J, Bleo+PBS group), accompanied by increased gene expression of lung Foxp3 (K, Bleo+PBS group) and IL‐10 (L, Bleo+PBS group). This effect was further strengthened by treatment with hAMSCs, that increased lung expression of Foxp3 (K, days 4 and 7) and of IL‐10 (L, days 7 and 9). Alveolar levels of T lymphocytes were analyzed by flow cytometry and expressed as percentage of viable CD45 positive cells in bronchoalveolar lavage collected from each mouse. Data from mRNA levels analysis are expressed as log_2_ of the fold change from the saline‐instilled group. These data are reported as mean ± SE of n = 4 (day 4) and n = 5‐7 (days 7, 9, and 14) samples. *P < .05, **P < .01 compared to control (Bleo+PBS) group

Notably, hAMSC treatment did not affect neither the levels of resident (data not shown) and Mo‐Alv macrophages (Figure 3A), nor that of CD11b^+^ DCs (Figure 2B). Instead, it reduced the percentage of Mo‐Alv macrophages (Figure 2C) and of CD11b^+^ DCs (Figure 2D) expressing the costimulatory molecule CD80 and decreased also the density of CD80 expression on their membranes (Figure 2E,F).

With regard to distinct macrophage subsets, we found that bleomycin challenge increased lung gene expression of the pan macrophagic marker F4/80 and of the markers associated with macrophage polarization toward M1 (iNOS) and M2 (Ym‐1) phenotype. As shown in Figure 2, hAMSC treatment did not alter the expression of F4/80 (Figure 2G) or of iNOS (Figure 2H) in lung tissue, whereas it induced the expression of Ym‐1 at days 4 and 7 (Figure 2I).

Altogether, these data suggest that hAMSC treatment promotes a shift toward immune phenotypes with lower inflammatory potential by decreasing CD80 expression and density on macrophages and dendritic cells, and by increasing expression of macrophage M2 markers.

### 3.3 hAMSCs alter T lymphocyte subsets in bleomycin‐induced inflammation

We also studied the ability of hAMSCs to counteract bleomycin‐induced alterations in alveolar and lung T‐cell environment.

Alveolar CD3^+^ T lymphocytes, which represent only 1.9% ± 0.9% of the total CD45^+^ population in saline‐instilled mice; increased to 13.9% ± 3.0% (day 4) and to 47.9% ± 8.1% (day 14) in bleomycin‐instilled animals (Figure 3A), with higher percentage of CD4^+^ cells compared to CD8^+^ cells (Figure 3B,C). hAMSC treatment did not reduce T lymphocyte recruitment, rather at day 4 it slightly increased the early recruitment of CD3^+^ cells, including both CD4^+^ and CD8^+^ subpopulations (Figure 3A‐C).

To reveal whether hAMSC treatment differently affects distinct inflammatory subsets of CD4^+^ T cells, we determined the lung gene expression of markers associated to Th1 (Tbet), Th2 (GATA3), and Th17 (ROR‐γτ) T‐cell subsets and of cytokines with proinflammatory action (IFN‐γ, IL‐1β, and IL‐4). As shown in Figure 3, bleomycin transiently increased Tbet and ROR‐γτ lung expression while GATA3 expression was high at all time points, suggesting an early role of Th1 and Th17 cells in contrast to a more consistent presence of Th2 cells in bleomycin‐challenged lungs (Figure 3D‐F). In line with the increased recruitment of CD3^+^ cells in alveolar spaces, we found that hAMSCs transiently induced Th1 and Th2 responses as indicated by the increased expression of Tbet at day 4 (Figure 3D) and of GATA‐3 at day 7 (Figure 3E). Instead, they transiently reduced Th17 response, as indicated by declined expression of ROR‐γτ at day 4 (Figure 3F). Moreover, the treatment with hAMSCs decreased the expression of IL‐1β (Figure 3G) while increased the expression of IL‐4 (Figure 3H). The treatment did not affect IFN‐γ expression (Figure 3I).

We also investigated the ability of hAMSCs to promote an immune suppressive milieu, by detecting the levels of alveolar T regulatory (Treg) cells, lung expression of the Treg marker Foxp3 and of IL‐10, a cytokine with important anti‐inflammatory activity. hAMSC treatment did not consistently affect alveolar Treg cell levels (Figure 3J), however it augmented expression of Foxp3 at days 4 and 7 (Figure 3K) and of IL‐10 at days 7 and 9 (Figure 3L) in lungs.

It is of note that hAMSC treatment did not affect neutrophil alveolar recruitment, whose relative levels transiently peaked at day 4 after bleomycin instillation (data not shown).

### 3.4 hAMSCs reduce bleomycin‐induced recruitment and maturation of B cells

**FIGURE 4:**
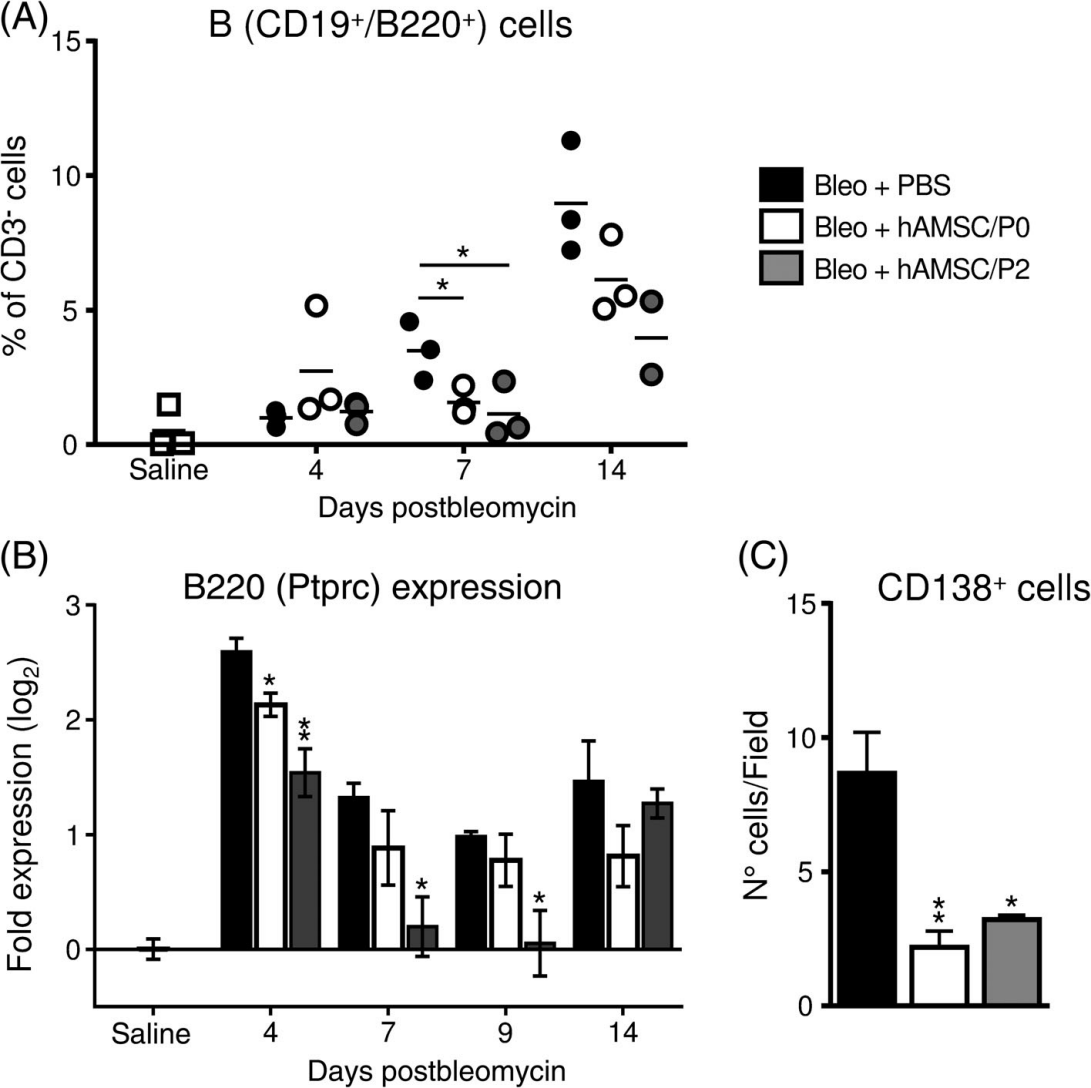
Human amniotic mesenchymal stromal cells (hAMSCs) reduce bleomycin‐induced recruitment and maturation of B cells. Bleomycin instillation produced a progressive recruitment of B cells in alveolar spaces (A, Bleo+PBS group) and increased mRNA expression of B220 (a marker specifically expressed by B lymphocytes) in lung tissue (B, Bleo+PBS group). hAMSC treatment markedly decreased B‐cell levels in alveolar spaces (A) and lung expression of B‐cell marker B220 (B). hAMSC treatment also decreased the number of CD138 positive cells (antibody‐secreting B cells) in lung tissues (C), suggesting that treatment control the maturation of B cells. Alveolar levels of B cells were analyzed by flow cytometry as percentage of CD3 negative cells in bronchoalveolar lavage collected from bleomycin‐challenged mice treated with amniotic cells (Bleo+hAMSC/P0 and Bleo+hAMSC/P2) or not (Bleo+PBS). Data from mRNA levels analysis (B) are expressed as log_2_ of the fold change from the saline‐instilled group. These data are reported as mean ± SE of n = 4 (day 4) and n = 5‐7 (days 7, 9, and 14) samples. CD138 positive cells were counted in whole lung sections derived from mice of all treatment groups, at 14 days from bleomycin challenge. *P < .05, **P < .01 compared to control (Bleo+PBS) group

To refine the picture of the inflammatory environment in our mouse model of fibrosis, we next focused on the effects exerted by bleomycin instillation and hAMSC treatment on B cells. As observed with myeloid cells and T lymphocytes, bleomycin challenge also induced recruitment of CD19^+^/B220^+^ B lymphocytes in alveolar spaces (Figure 4A) and upregulation of gene expression of the B‐cell marker B220 in lung tissues (Figure 4B). hAMSC treatment reduced the relative amount of alveolar B cells at day 7 (Figure 4A), and this effect was accompanied by a decline in the expression of B220 in lung tissue (Figure 4B). Remarkably, this process was not transient, but it extended to the fibrotic phase (day 14) of bleomycin‐induced injury (Figure 4B).

Lastly, hAMSC treatment also reduced the number of CD138^+^ antibody‐secreting cells present in lung tissues of bleomycin‐instilled mice (Figure 4C), suggesting an impairment of the maturation of B cells toward antibody‐secreting cells.

### 3.5 hAMSCs compromise the formation and expansion of bleomycin‐induced lymphoid aggregates

Given that intrapulmonary B‐cell aggregates have been found in lungs of patients with inflammatory/fibrotic respiratory diseases,^4^ we evaluated B lymphocyte distribution in lungs from bleomycin‐challenged mice.

**FIGURE 5:**
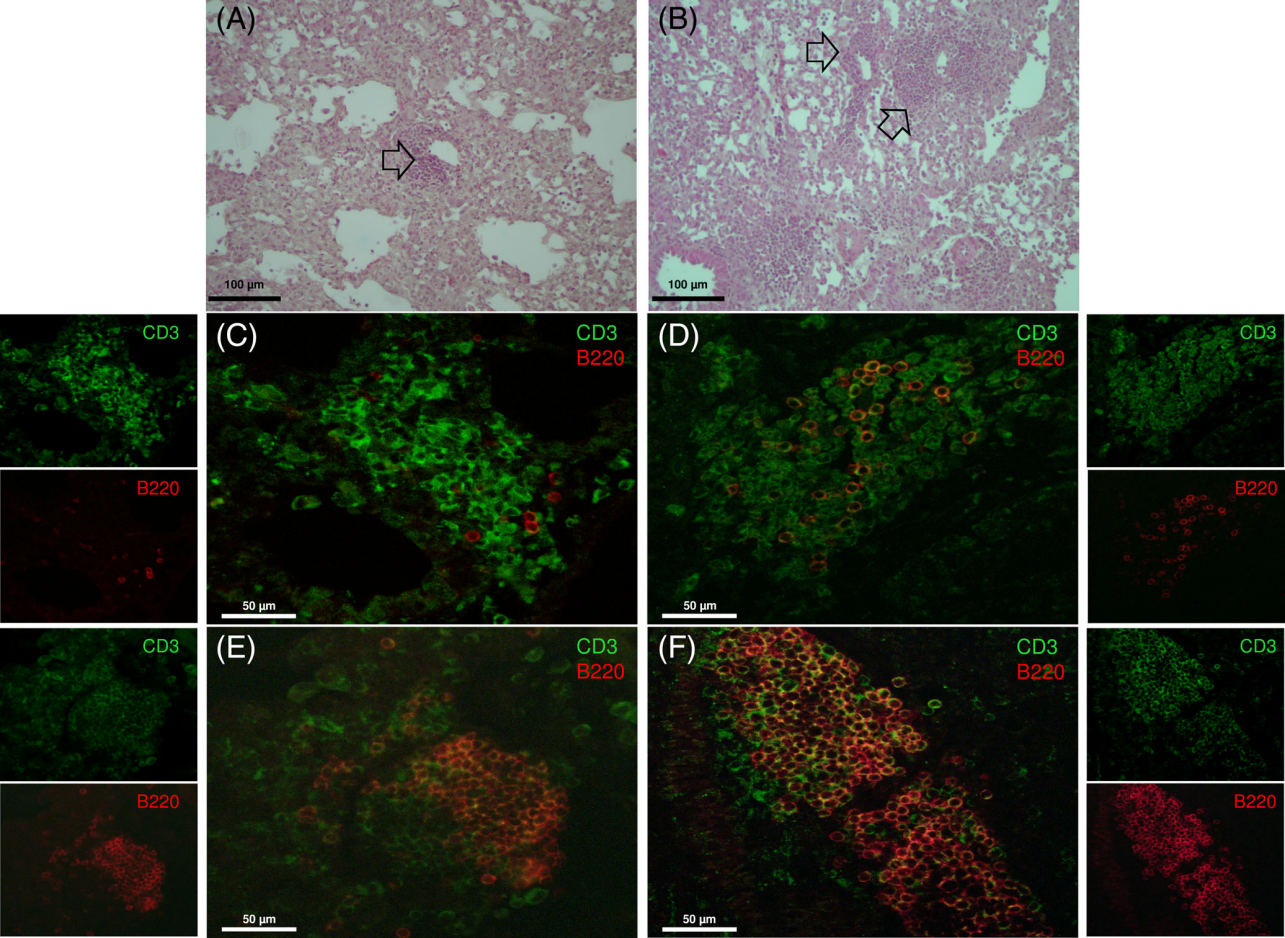
Human amniotic mesenchymal stromal cells (hAMSCs) reduce the presence of lymphoid aggregates in lung tissues of bleomycin‐challenged mice. Lymphoid aggregates (indicated with black arrows) were present in the lungs of bleomycin‐challenged mice. They were located in peri‐bronchial areas as shown in lung sections stained with hematoxylin and eosin (A,B). Immunofluorescence double staining for CD3 (T cells, green) and B220 (B cells, red) indicated that lung lymphoid aggregates are composed of T and B lymphocytes mixed in variable proportions (C‐F). Here, examples of aggregates with low (C,D), intermediate (E), and large (F) presence of B cells are reported. Scale bars = 100 μm (A,B); 50 μm (C‐F)

Hematoxylin and eosin analysis of lung sections evidenced the presence of numerous lymphoid aggregates mainly located within the peri‐bronchial areas (Figure 5A,B, arrows). These aggregates were primarily composed of CD3^+^ T and B220^+^ B lymphocytes mixed in variable proportions (Figure 5C‐F).

**FIGURE 6:**
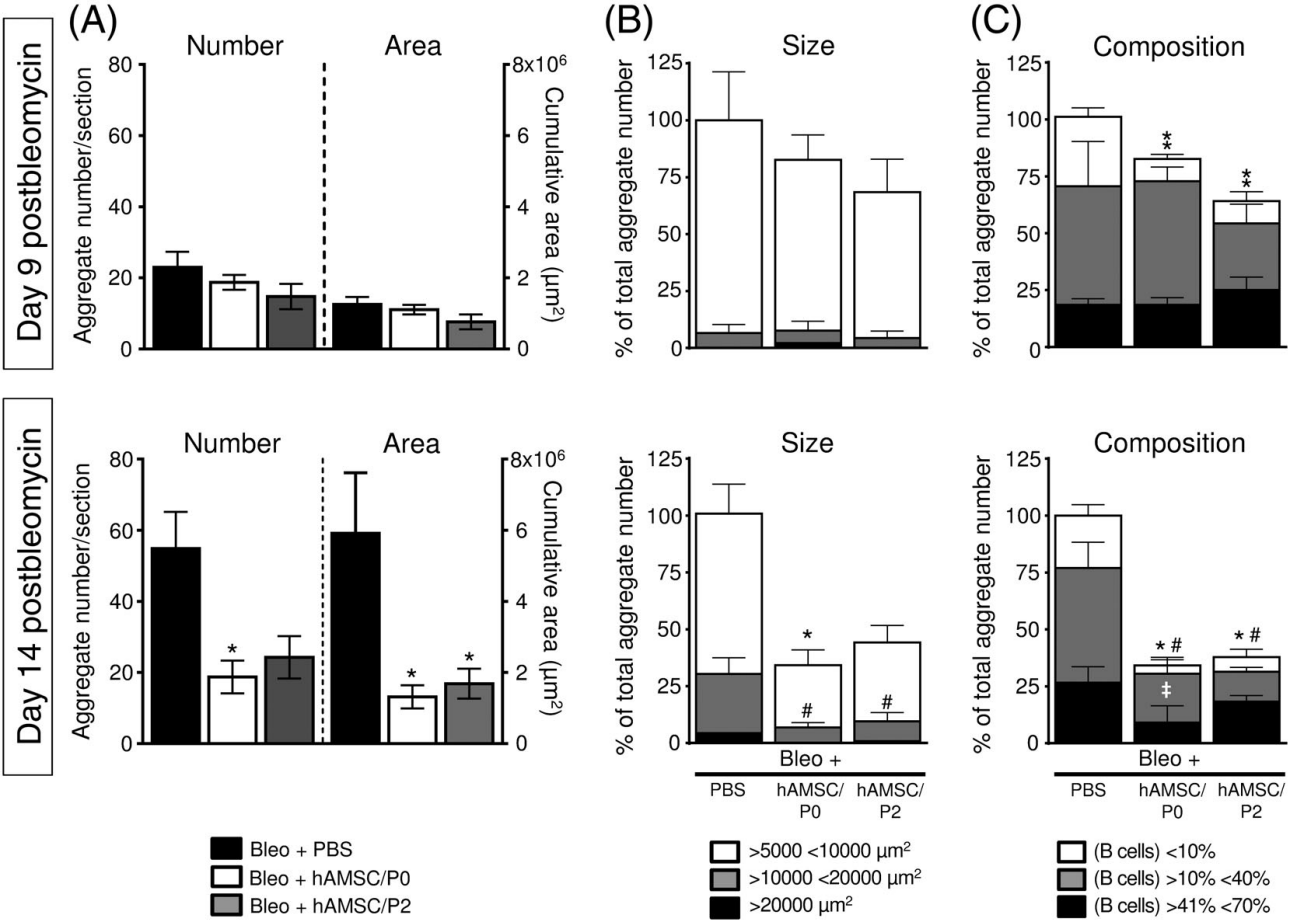
Human amniotic mesenchymal stromal cells (hAMSCs) compromise the formation and expansion of bleomycin‐induced lymphoid aggregates. Bleomycin instillation induced the formation of lymphoid aggregates in lung tissue. The number and the cumulative area of aggregates increased from days 9 to 14 postbleomycin (A). hAMSC treatment inhibited the formation of new aggregates and blocked their expansion, indeed the number and the cumulative areas of aggregates in hAMSC‐treated mice were stable from days 9 to 14 (A). In control mice (Bleo+PBS), at day 9 postbleomycin most of the single aggregates had a size ranging from 5000 to 10 000 μm^2^; at day 14 their size increased and aggregates with an intermediate (from 10 000 to 20 000 μm^2^) and large size (more than 20 000 μm^2^) developed/appeared (B). hAMSC treatment almost abolished the expansion of single aggregates, indeed a lower number of small and intermediate aggregates and very few large aggregate were detected (B; Bleo+hAMSC/P0 and Bleo+hAMSC/P2 groups). Control mice (Bleo+PBS) had lymphoid aggregates composed of T and B lymphocytes in variable proportions (C). At days 9 and 14, lymphoid aggregates with an intermediate presence of B cells (10%‐40% with respect to the total lymphoid population), represented approximately 75% of the total aggregates (C, gray bars). Between days 9 and 14, the aggregates with a higher presence of B cells (from 41% to 70%) increased (C, black bars). hAMSC treatment prevented the enrichment in B cells in lung lymphoid aggregates (C; Bleo+hAMSC/P0 and Bleo+hAMSC/P2 groups). Data are reported as mean ± SE of n = 4 (for control and treated groups at day 9) and n = 5 (for control and treated groups at day 14). A, *P < .05 compared to control (Bleo+PBS) group. B, *P < .05 by comparing number of aggregates with small size (>5000 <10 000 μm^2^) between control (Bleo+PBS) group and treated groups (Bleo+hAMSC/P0 and Bleo+hAMSC/P2). #*P* < .05 by comparing number of aggregates with intermediate size (>10 000 <20 000 μm^2^). C, *P < .05, **P < .01 by comparing number of aggregates with low presence of B cells (<10 %) between control (Bleo+PBS) group and treated groups (Bleo+hAMSC/P0 and Bleo+hAMSC/P2). #*P* < .05 by comparing number of aggregates with intermediate presence of B cells (>10% <40%). ‡*P* < .05 by comparing number of aggregates with large presence of B cells (>41% <70%)

In bleomycin‐treated animals, between days 9 and 14 postinstillation, the number and the cumulative area of lung lymphoid aggregates markedly increased, ranging from 23.0 ± 4.3 per section and 1.26 × 10^5^ ± 0.02 × 10^5^ μm^2^ at day 9, to 54.8 ± 10.4 per section and 5.91 × 10^5^ ± 1.69 × 10^5^ μm^2^ at day 14 (Figure 6A). Interestingly, over this time, hAMSC treatment significantly prevented the formation of new aggregates and blocked the increment of cumulative area (Figure 6A).

Next, we examined the ability of hAMSCs to influence the expansion of single lymphoid aggregates. At day 9, most of the aggregates ranged from 5000 to 10 000 μm^2^ (Figure 6B, upper panel), whereas at day 14 more than 25% of the aggregates had an intermediate size (from 10 000 to 20 000 μm^2^) and some bigger aggregates appeared (higher than 20 000 μm^2^; Figure 6B, lower panel). hAMSC treatment almost abolished the expansion of single lymphoid aggregates at day 14, as indicated by the presence of only few small and intermediate aggregates and very few large aggregates (Figure 6B, lower panel).

Finally, we explored whether hAMSCs affected the cellular composition of the aggregates.

At days 9 and 14, lymphoid aggregates with an intermediate presence of B cells (10%‐40% with respect to the total cell lymphoid population), represented approximately 75% of the total aggregates (Figure 6C, gray bars). Between days 9 and 14, the aggregates with a higher presence of B cells (from 41% to 70%) increased from 18.5% ± 2.3% (at day 9) to 26.6% ± 5.2% (Figure 6C, black bars). Interestingly, at day 14, hAMSC treatment reduced the number of B cells into the lymphoid aggregates (Figure 6C). Overall, these data indicate that hAMSCs blocked the formation, expansion, and B‐cell enrichment of lung lymphoid aggregates.

To provide insight onto how hAMSCs affected B‐cell lymphoid aggregates, we investigated B‐cell recruitment, survival, and proliferation after treatment.

**FIGURE 7:**
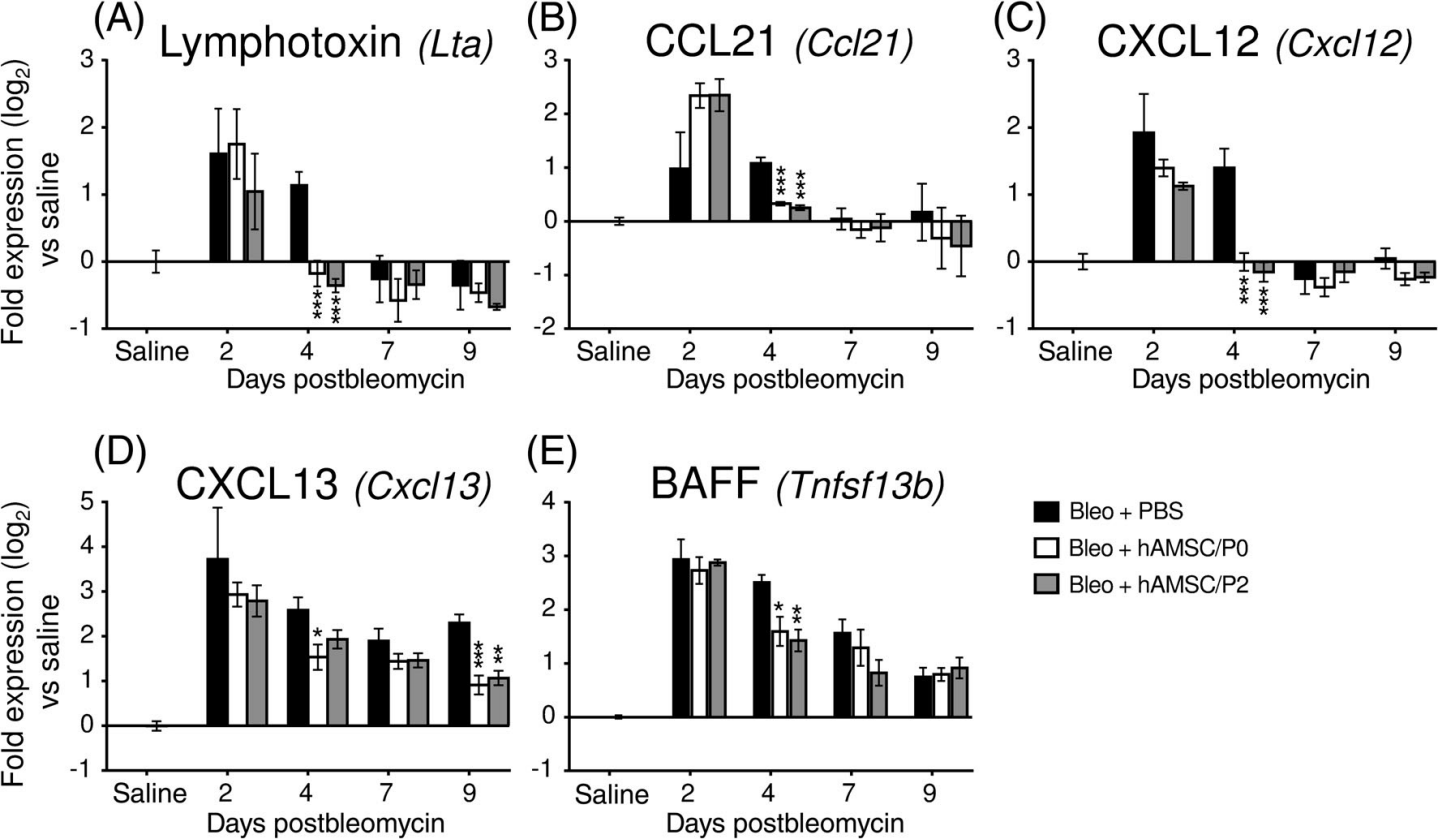
Human amniotic mesenchymal stromal cell (hAMSC) treatment reduces the expression of chemokines/cytokines involved in pulmonary recruitment and retention of B lymphocytes. Compared to saline‐instilled mice, bleomycin challenge upregulated the gene expression of lymphotoxin (A, Bleo+PBS group), CCL21 (B), CXCL12 (C), and CXCL13 (D), which promote the recruitment and retention of lymphocytes into lung tissue. Expression of lymphotoxin, CCL21, and CXCL12 were early and transiently increased, whereas CXCL13 expression (the most effective chemoattractant for B cells) was high at all time points. hAMSC treatment accelerated the normalization of expression of lymphotoxin, CCL21 and CXCL12, and reduced CXCL13 expression at all time points. In addition, bleomycin challenge increased BAFF gene expression (E, Bleo+PBS group), a cytokine with a crucial role for B‐cell survival. hAMSC treatment reduced BAFF expression at days 4 and 7 (E). Data are expressed as log_2_ of the fold change expression from the saline‐instilled group, and are reported as mean ± SE of n = 3 (day 2), n = 4 (day 4), and n = 5‐7 (days 7 and 9). *P < .05, **P < .01, ***P < .01 compared to control (Bleo+PBS) group

To investigate whether hAMSCs can affect the recruitment of lymphocytes in lung tissues, we performed gene expression analysis of the main chemokines involved in recruitment and retention of lymphocytes (lymphotoxin, CCL21, CXCL12, and CXCL13), and known to contribute to the generation of lymphoid aggregates in various organs.^34^, ^35^ As indicated in Figure 7, bleomycin treatment transiently increased the expression of lymphotoxin, CCL21, and CXCL12 (Figure 7A‐C), while CXCL13, previously suggested to be a potent chemokine for B cells,^36^ remained highly expressed at all time points (Figure 7D). By day 4, hAMSC treatment reduced lung expression of all the chemokines examined (Figure 7A‐D).

To inquire into the ability of hAMSCs to interfere with the production and/or secretion of factors crucial for B‐cell survival, we analyzed the expression of A proliferation‐inducing ligand (April) and B‐cell‐activating factor (BAFF). Neither bleomycin nor hAMSC treatment altered the expression of April in lung tissue (data not shown). On the other hand, bleomycin upregulated BAFF expression at all time points and hAMSCs reduced its expression at day 4 (Figure 7E).

We next sought to ascertain the effect of hAMSCs on B‐cell proliferation. Analysis of Ki67 positive cells revealed that only a small number of B cells present in lymphoid aggregates proliferate (data not shown), thus suggesting that most of B cells may be recruited from extrapulmonary compartments.

## 4 DISCUSSION

In this study, we investigated if amniotic mesenchymal stromal cells (hAMSCs) isolated from human term placenta interfere with the inflammatory response in a mouse model of pulmonary fibrosis. For the first time, we demonstrate the ability of hAMSCs to impair lung B‐cell response and we propose this as a possible mechanism crucial to reduce the progression of fibrotic lesions.

In agreement with ours and others' previous studies performed in bleomycin‐induced and in other models of fibrosis,^7^, ^8^, ^9^, ^10^, ^18^, ^24^ our data confirm the ability of hAMSCs to reduce fibrosis development and demonstrate their efficiency in lowering the inflammatory milieu created by bleomycin instillation. hAMSCs, by reducing costimulatory protein expressions in dendritic cells and in monocyte‐derived macrophages, possibly reduce their ability to induce antigen‐specific T‐cell immune responses. Moreover, hAMSCs promote the generation of immune cells (Treg) and cytokines (IL‐10) with immune regulatory functions in diseased lungs.

Importantly, our data show that hAMSCs affect the kinetics of B‐cell recruitment and B‐cell homing throughout the entire fibrotic process, from the early inflammatory phase (days 2‐7) to the late fibrotic phase (days 7‐14) of bleomycin‐induced lung injury. We found indeed that hAMSC treatment constantly reduced the relative amount of B cells in alveolar spaces and lung expression of B220, a murine B‐cell marker, suggesting this as a possible mechanism of hAMSCs to contrast lung fibrosis. Moreover, our data underline the potential involvement of B cells in the pathogenesis of bleomycin‐induced fibrotic lesions already proposed by some authors. Komura and collaborators^37^ reported that genetically modified mice lacking B‐cell surface molecule CD19 displayed a reduced susceptibility to bleomycin‐induced fibrosis. Francois and collaborators^38^ observed that genetic ablation of BAFF attenuates pulmonary fibrosis and O'Donoghue and collaborators^39^ detected reduced fibrosis in mutant mice deficient in mature B lymphocytes, challenged with bleomycin.

In addition, the presence of lymphoid aggregates has been described in the lungs of mice challenged with bleomycin,^40^ of mice genetically modified (CCR7−/− mice) showing an impaired homing of Treg cells,^41^ and of mice with rheumatoid arthritis‐related interstitial lung disease.^42^

Here, we also detected intrapulmonary aggregates composed of T and B lymphocytes in bleomycin‐instilled animals and we demonstrated that hAMSC treatment inhibited the formation and expansion of these aggregates.

Although the role of lung lymphoid aggregates is controversial, a growing body of clinical evidence strongly points at the contribution of intrapulmonary T/B‐cell aggregates in the initiation and/or progression of a range of fibrotic diseases such as IPF^1^, ^2^, ^43^, ^44^ and nonspecific interstitial pneumonia.^45^ In the lungs of IPF patients, B cells form focal aggregates together with T lymphocytes and their number has been correlated with the development and severity of lung fibrotic lesions.^2^, ^45^ In addition, as observed herein, B lymphocytes present in these aggregates do not proliferate,^1^, ^2^, ^40^, ^43^ suggesting that recruitment from systemic circulation likely represents the main mechanism leading to lymphocyte accumulation in lung parenchyma.

Importantly, our data suggest that hAMSC treatment can reduce B‐cell recruitment and homing by inhibiting the lung expression of homeostatic lymphoid chemokines, essentially CXCL13, a crucial chemoattractant for B cells,^36^ and by reducing BAFF, that holds a major role in B‐cell survival and maturation and can thereby promote lymphoid aggregate formation.^46^ It is of note that in IPF patients, increased plasma concentrations of BAFF (or BLyS) correlated with clinical severity and negative outcomes of these patients^43^ and longitudinal increment of plasma levels of CXCL13 and high lung expression of CXCL13 have been associated with severe clinical manifestations and disease progression.^44^

Besides B and T cells and their mediators, other immune cells may participate in supporting the formation and expansion of intrapulmonary lymphoid aggregates. Dendritic cells, for example, have been suggested to be crucial in maintaining lymphoid aggregates in lungs from influenza virus‐infected mice, both by providing lymphotoxin and CXCL13 necessary to B‐cell homing and retention and by promoting T‐cell activation.^47^ Moreover, macrophages represent a potent inducible source of CXCL13 in chronic inflammatory diseases associated with formation of T‐ and B‐cell aggregates, such as rheumatoid arthritis and ulcerative colitis, where they possibly play a role in the genesis of the lymphoid tissue.^48^ Considering that hAMSC treatment impaired the antigen presenting potential of dendritic cells and monocyte‐derived macrophages, we hypothesize that this also may contribute to the ability of hAMSCs in controlling lymphoid aggregate formation/expansion and fibrosis progression in bleomycin‐injured lungs.

Altogether, we propose that in bleomycin‐challenged mice, hAMSCs create an anti‐inflammatory microenvironment partially mediated by their ability to control recruitment, retention, and maturation of B cells in diseased lungs. Within the lymphoid aggregates, B cells can continuously act as antigen presenting cells for the adjacent T lymphocytes and can cause cytotoxicity by producing autoantibodies. hAMSCs can resolve this loop and hence break the self‐maintaining inflammatory condition promoted by B cells.^43^, ^44^, ^49^

Finally, in an attempt to support the clinical application of hAMSC‐based therapy, this study tried to address the important question on the impact of in vitro expansion on hAMSC therapeutic effects. In this study, indeed two different cell preparations were used: freshly isolated hAMSCs (hAMSC/P0) and hAMSCs expanded in vitro to passage 2 (hAMSC/P2). In bleomycin‐induced pulmonary injury model, both treatments displayed similar effects on the immune populations investigated in this study, suggesting that at least a short‐term expansion in vitro does not alter cell activity.

## 5 CONCLUSION

Our work provides key insights into the therapeutic potential of hAMSCs from an immunological perspective, providing further evidence for a potential clinical translation of hAMSCs in inflammation‐related fibrotic diseases. In support of this, short‐term in vitro expansion (passage 2) did not alter the activity of hAMSCs in comparison to freshly isolated cells (passage 0), thus further encouraging the translation of this therapeutic product into the clinic. However, given the need of high numbers of cells also for potential repeated treatments, studies elucidating the influence of in vitro long‐term expansion on cellular functions are needed.

## CONFLICT OF INTEREST

O.P. is inventor of intellectual property (patents US8524283B2 and EP2171042B1). The other authors indicated no financial relationships.

## AUTHOR CONTRIBUTIONS

A.C.: conception and design, collection of data, data analysis and interpretation, and manuscript writing; P.R.: performed animal experiments and prepared hAMSCs from amniotic membranes; P.B.S.: conducted molecular analysis; S.F.: performed immunohistochemical and immunofluorescence analysis; M.M., E.V.: performed flow cytometry analysis; I.T.: responsible for animal care; V.C.: collaboration for animal care; A.R.S., F.R.S.: manuscript drafting and editing; O.P.: conception and design, financial support, data analysis and interpretation, manuscript writing, and final approval of manuscript.

## Supporting information


**Table S1** Primer sequences for RT‐PCR

**Table S2** CD45^+^ cell count in BAL

**Figure S1** Gating strategy applied in flow‐cytometric analysis

## Data Availability

All relevant data are available from the corresponding author upon reasonable request.
