# Supplementary material for: Amniotic MSCs reduce pulmonary fibrosis by hampering lung B‐cell recruitment, retention, and maturation
Source: Stem Cells Transl Med. 2020 May 26;9(9):1023–35. doi: 10.1002/sctm.20-0068 (PMC7445028; doi:10.1002/sctm.20-0068)
Supplement: Supplementary file 1 — Table S1 Primer sequences for RT‐PCR [file SCT3-9-1023-s001.docx]

**Table S1.** Primer sequences for RT-PCR

| **Genes** | **Forward primer sequence (5'-3')** | **Reverse primer sequence (5'-3')** |
| --- | --- | --- |
|  |  |  |
| *Pdpn* (Podoplanin) | CACCTCAGCAACCTCAGAC | ACAGGGCAAGTTGGAAGC |
|  |  |  |
| *Acta2* (α-SMA) | GAGGCACCACTGAACCCTAA | CATCTCCAGAGTCCAGCACA |
|  |  |  |
| *Fn1* (Fibronectin) | CGAGGTGACAGAGACCACAA | CTGGAGTCAAGCCAGACACA |
|  |  |  |
| *Col1a1 (*Collagen 1A1) | GATGACGTGCAATGCAATGAA | CCCTCGACTCCTACATCTTCTGA |
|  |  |  |
| *Cd4* (CD4) | GAGAGTCAGCGGAGTTCT | CTCACAGGTCAAAGTATTGTT |
|  |  |  |
| *Tbx21* (T-bet) | CAAGTGGGTGCAGTGTGGAAAG | TGGAGAGACTGCAGGACGATC |
|  |  |  |
| *Gata3* (GATA-3) | TCTCACTCTCGAGGCAGCATGA | GGTACCATCTCGCCGCCACAG |
|  |  |  |
| *Rorc* (ROR-γτ) | ACGGCCCTGGTTCTCATCA | CCAAATTGTATTGCAGATGTTCCAC |
|  |  |  |
| *Foxp3* (Fox-p3) | CACCCAGGAAAGACAGCAACC | GCAAGAGCTCTTGTCCATTGA |
|  |  |  |
| *Ptprc* (CD45R/B220) | AATGGCTCTTCAGAGACCACATA | AGTCAGGCTGTGGGGACA |
|  |  |  |
| *Adgre1* (F4/80) | CTTTGGCTATGGGCTTCCAGT | GCAAGGAGGACAGAGTTTATCGTG |
|  |  |  |
| *Nos2* (iNOS) | CTGCAGCACTTGGATCAGGAACCTG | GGAGTAGCCTGTGTGCACCTGGAA |
|  |  |  |
| *Chil3* (Ym-1) | GGCTACACTGGAGAAAATAGTCCC | CCAACCCACTCATTACCCTGATAG |
|  |  |  |
| *Il10* (IL-10) | GGACAACATACTGCTAACCGAC | AAAATCACTCTTCACCTGCTCC |
|  |  |  |
| *Il1b* (IL-1β) | TGTAATGAAAGACGGCACACC | TCTTCTTTGGGTATTGCTTGG |
|  |  |  |
| *Ifng* (IFN-γ) | TCAAGTGGCATAGATGTGGAAGAA | TGGCTCTGCAGGATTTTCATG |
|  |  |  |
| *Il4* (IL-4) | CGAAGAACACCACAGAGAGTGAGC T | GACTCATTCATGGTGCAGCTTATCG |
|  |  |  |
| *Lta* (Lymphotoxin) | GCTTGGCACCCCTCCTGTC | GATGCCATGGGTCAAGTGCT |
|  |  |  |
| *Ccl21* (CCL21) | CCCCTGGACCCAAGGCAGTGA | TTGCCGGGATGGGACAGCCT |
|  |  |  |
| *Cxcl12* (CXCL12) | GCGCTCTGCATCAGTGACGGTAA | GCTTGACGTTGGCTCTGGCGA |
|  |  |  |
| *Cxcl13* (CXCL13) | CATAGATCGGATTCAAGTTACGCC | TCTTGGTCCAGATCACAACTTCA |
|  |  |  |
| *Tnfsf13* (April) | GGTGGTATCTCGGGAAGGAC | CCCCTTGATGTAAATGAAAGACA |
|  |  |  |
| *Tnfsf13b* (BAFF) | CAGGAACAGACGCGCTTTC | GTTGAGAATGGCGGCATCC |
|  |  |  |
| *Actb* (β-actin) | GCAGCTCAGTAACAGTCCGC | AGTGTGACGTTGACATCCGT |
|  |  |  |
