# Supplementary material for: Amniotic MSCs reduce pulmonary fibrosis by hampering lung B‐cell recruitment, retention, and maturation
Source: Stem Cells Transl Med. 2020 May 26;9(9):1023–35. doi: 10.1002/sctm.20-0068 (PMC7445028; doi:10.1002/sctm.20-0068)
Supplement: Supplementary file 2 — Table S2 CD45+ cell count in BAL [file SCT3-9-1023-s002.docx]

Table S2. CD45^+^ cell count in BAL

| Treatment group | Days post bleomycin instillation | | |
| --- | --- | --- | --- |
|  | 4 | 7 | 14 |
|  |  |  |  |
| Bleo + PBS | 47869 ± 7008 | 162267 ± 17897 | 215635 ± 52662 |
|  |  |  |  |
| Bleo + hAMSC/P0 | 81178 ± 16747 | 170967 ± 49798 | 29198 5± 54642 |
|  |  |  |  |
| Bleo + hAMSC/P2 | 79788 ± 7232 | 135731 ± 34825 | 231369 ± 25926 |
|  |  |  |  |

Count of CD45^+^ cells by flow-cytometryin BAL collected from control untreated (Bleo+PBS) and treated (Bleo+hAMSC/P0 and Bleo+h AMSC/P2) groups at different time points from bleomycin instillation.
