# Supplementary material for: Amniotic MSCs reduce pulmonary fibrosis by hampering lung B‐cell recruitment, retention, and maturation
Source: Stem Cells Transl Med. 2020 May 26;9(9):1023–35. doi: 10.1002/sctm.20-0068 (PMC7445028; doi:10.1002/sctm.20-0068)
Supplement: Supplementary file 3 — Figure S1 Gating strategy applied in flow‐cytometric analysis [file SCT3-9-1023-s003.pdf]

Amniotic MSC reduce pulmonary fibrosis by hampering lung B cell recruitment, retention and maturation

Anna Cargnoni[1], Pietro Romele[1], Patrizia Bonassi Signoroni[1], Serafina Farigu[1], Marta Magatti[1], Elsa Vertua[1], Ivan Toschi[2], Valentina Cesari[2], Antonietta R. Silini[1], Francesca R. Stefani[1], Ornella Parolini[1,3]

SUPPLEMENTAL INFORMATION

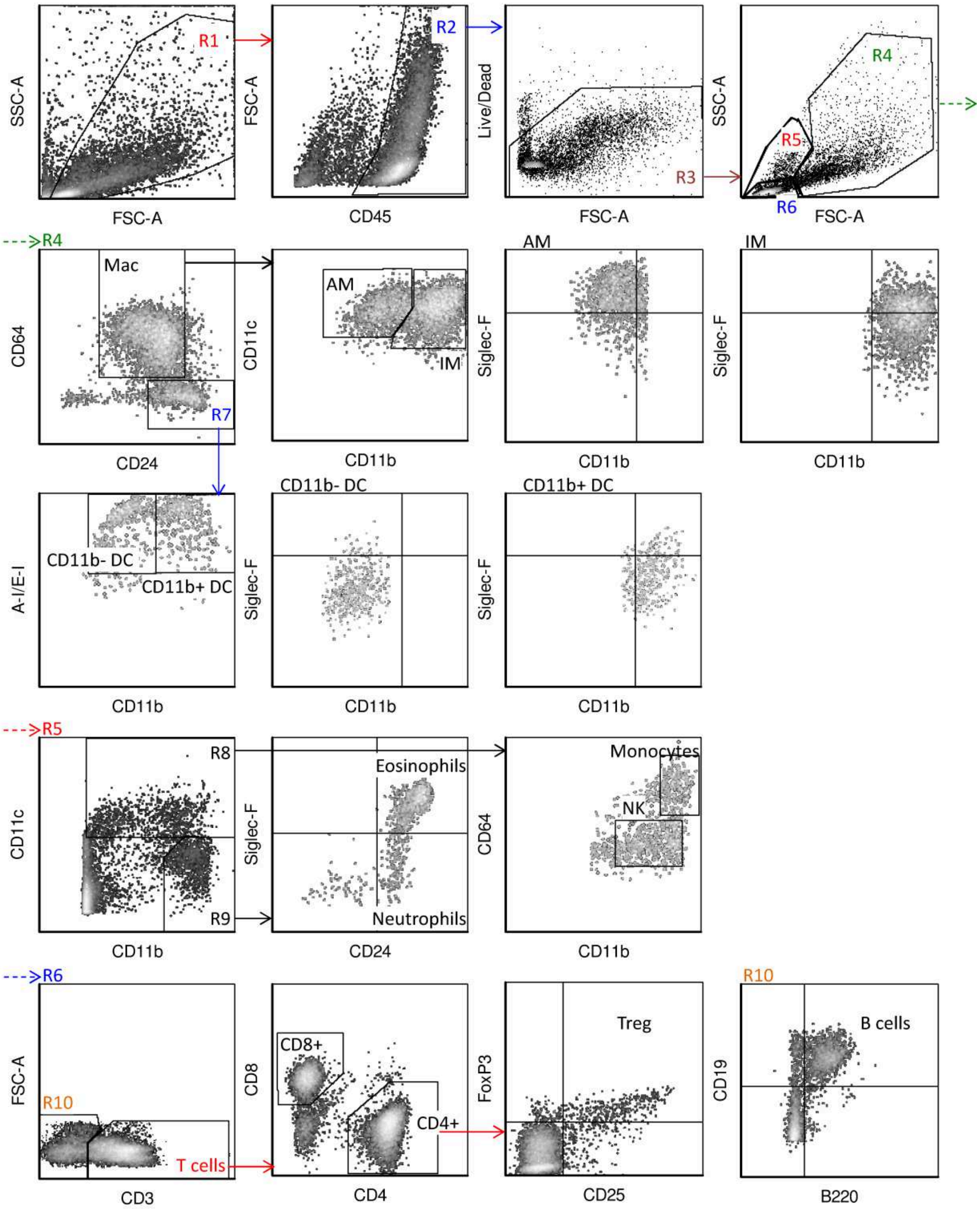

Figure-1S: Gating strategy applied in flow-cytometric analysis

Table S1. Primer sequences for RT-PCR

Genes	Forward primer sequence (5'-3')	Reverse primer sequence (5'-3')
Pdpn (Podoplanin)	CACCTCAGCAACCTCAGAC	ACAGGGCAAGTTGGAAGC
Acta2 (α-SMA)	GAGGCACCACTGAACCCTAA	CATCTCCAGAGTCCAGCACA
Fn1 (Fibronectin)	CGAGGTGACAGAGACCACAA	CTGGAGTCAAGCCAGACACA
*Col1a1 (*Collagen 1A1)	GATGACGTGCAATGCAATGAA	CCCTCGACTCCTACATCTTCTGA
Cd4 (CD4)	GAGAGTCAGCGGAGTTCT	CTCACAGGTCAAAGTATTGTT
Tbx21 (T-bet)	CAAGTGGGTGCAGTGTGGAAAG	TGGAGAGACTGCAGGACGATC
Gata3 (GATA-3)	TCTCACTCTCGAGGCAGCATGA	GGTACCATCTCGCCGCCACAG
Rorc (ROR-γτ)	ACGGCCCTGGTTCTCATCA	CCAAATTGTATTGCAGATGTTCCAC
Foxp3 (Fox-p3)	CACCCAGGAAAGACAGCAACC	GCAAGAGCTCTTGTCCATTGA
Ptprc (CD45R/B220)	AATGGCTCTTCAGAGACCACATA	AGTCAGGCTGTGGGGACA
Adgre1 (F4/80)	CTTTGGCTATGGGCTTCCAGT	GCAAGGAGGACAGAGTTTATCGTG
Nos2 (iNOS)	CTGCAGCACTTGGATCAGGAACCTG	GGAGTAGCCTGTGTGCACCTGGAA
Chil3 (Ym-1)	GGCTACACTGGAGAAAATAGTCCC	CCAACCCACTCATTACCCTGATAG
Il10 (IL-10)	GGACAACATACTGCTAACCGAC	AAAATCACTCTTCACCTGCTCC
Il1b (IL-1β)	TGTAATGAAAGACGGCACACC	TCTTCTTTGGGTATTGCTTGG
Ifng (IFN-γ)	TCAAGTGGCATAGATGTGGAAGAA	TGGCTCTGCAGGATTTTCATG
Il4 (IL-4)	CGAAGAACACCACAGAGAGTGAGC T	GACTCATTCATGGTGCAGCTTATCG
Lta (Lymphotoxin)	GCTTGGCACCCCTCCTGTC	GATGCCATGGGTCAAGTGCT
Ccl21 (CCL21)	CCCCTGGACCCAAGGCAGTGA	TTGCCGGGATGGGACAGCCT
Cxcl12 (CXCL12)	GCGCTCTGCATCAGTGACGGTAA	GCTTGACGTTGGCTCTGGCGA
Cxcl13 (CXCL13)	CATAGATCGGATTCAAGTTACGCC	TCTTGGTCCAGATCACAACTTCA
Tnfsf13 (April)	GGTGGTATCTCGGGAAGGAC	CCCCTTGATGTAAATGAAAGACA
Tnfsf13b (BAFF)	CAGGAACAGACGCGCTTTC	GTTGAGAATGGCGGCATCC
Actb (β-actin)	GCAGCTCAGTAACAGTCCGC	AGTGTGACGTTGACATCCGT

Table S2. CD45^{+} cell count in BAL

Treatment group	Days post bleomycin instillation		
	4	7	14
Bleo + PBS	47869 ± 7008	162267 ± 17897	215635 ± 52662
Bleo + hAMSC/P0	81178 ± 16747	170967 ± 49798	29198 5± 54642
Bleo + hAMSC/P2	79788 ± 7232	135731 ± 34825	231369 ± 25926

Count of CD45^{+} cells by flow-cytometryin BAL collected from control untreated (Bleo+PBS) and treated (Bleo+hAMSC/P0 and Bleo+h AMSC/P2) groups at different time points from bleomycin instillation.
